# Who Takes Long Daytime Naps? Results of the ‘German Health Update’ (GEDA 2023)

**DOI:** 10.25646/14592

**Published:** 2026-11-04

**Authors:** Janina Reinmuth, Simon Löbl, Almut Richter, Julika Loss, Anja Schienkiewitz

**Affiliations:** 1 Robert Koch Institute, Department of Epidemiology and Health Monitoring, Berlin, Germany; 2 University of Heidelberg, Medical Faculty Mannheim, Centre for Preventive Medicine and Digital Health (CPD), Mannheim, Germany

**Keywords:** Napping, Sleep duration, Prevalence, Health surveys, Germany, Education, Gender

## Abstract

**Background:**

In the international literature, napping has been associated with positive health outcomes; however, naps lasting at least 60 minutes are linked to adverse health impacts. Until now, representative data on the frequency and duration of naps in Germany have been lacking.

**Method:**

Using data from the representative GEDA 2023 study (n = 7,967), this fact sheet presents the prevalence of naps and extended naps (≥ 60 minutes) for women and men, stratified by age and educational groups.

**Results:**

Almost half of the adult population in Germany takes a nap at least once a week, a quarter of whom for 60 minutes or more. Extended naps are more prevalent among women under 45 and among less educated men compared with their higher-educated peers.

**Conclusions:**

The results highlight the prevalence of napping in the German population, as well as the social groups that more frequently take extended naps and may therefore face higher health risks.

**Key messages**

► A daytime nap lasting at least 60 minutes is associated with an increased risk of e.g. cardiovascular disease, dementia and depression.
► 45.3 % of adults in Germany nap at least once a week: 47.0 % of women and 43.7 % of men.
► 25.5 % of people who nap at least once a week sleep for 60 minutes or longer.
► Women under 45 years more frequently take an extended nap.
► Men with higher formal education are less likely to take extended daytime naps (≥ 60 minutes) compared with men with lower formal education.

**GEDA 2023**

German Health Update (data collection 2023)

**Data holder:** Robert Koch Institute

**Objectives:** Provision of reliable information on the health status, health behaviour and health care of the population living in Germany

**Study design:** Cross-sectional telephone survey

**Population:** German-speaking population aged 18 and older living in private households that can be reached via landline or mobile phone

**Sampling:** Random sample of landline and mobile telephone numbers (dual-frame method) from the ADM sampling system (Arbeitskreis Deutscher Markt- und Sozialforschungsinstitute e. V.)

**Sample size:** Ca. 1,000 participants per questionnaire module per month

**Data collection period:** January 2023 – February 2024

GEDA survey waves: GEDA 2009, GEDA 2010, GEDA 2012, GEDA 2014/2015-EHIS, GEDA 2019/2020-EHIS

Continuous survey between 2021 and 2023

Further information is available at www.rki.de/geda-en

## 1. Introduction 

A daytime nap is generally understood as a short period of sleep during the day, typically occurring around midday and separate from night-time sleep [1]. A standardized definition of napping is lacking, leading to variations in how the frequency and duration of daytime sleep are measured across scientific studies [2]. When it comes to the health impacts of daytime napping, duration appears to be more crucial than frequency [1–3]. While a short nap of under 60 minutes is associated with positive physical and cognitive effects, such as reduced fatigue and improved performance, longer naps of at least 60 minutes correlate with health risks [4] such as an increased risk of metabolic disorders, cardiovascular disease, dementia and depression, as well as increased overall mortality [1, 5, 6]. Daytime napping may be common and routinely practiced among the adult population in Germany. A meta- analysis comprising cohort studies from various countries indicates an average napping prevalence of 44.5 % [3], consistent with findings from a German cohort study where almost 50 % of the population naps at least occasionally [7]. Although men nap more frequently, women indicate longer nap durations [8]. Furthermore, older people appear to take daytime naps more frequently [9].

Current, representative data on daytime naps in Germany are lacking. This analysis therefore aims to present the prevalence and duration of napping among adults in Germany. Particular focus is placed on naps lasting at least 60 minutes, as these are associated with adverse health consequences.

## 2. Methods

Data from the ‘German Health Update’ (GEDA) study from 2023 (data set version v3) are used to describe the frequency and duration of napping among adults in Germany (Infobox). GEDA is a telephone-based cross-sectional survey conducted by the Robert Koch Institute that is representative of the population living in private households in Germany; it collects data on health status and selected influencing factors [10]. Questions on sleep behaviour were collected in GEDA 2023 between May 2023 and February 2024 (n = 8,026). For the cross-sectional analyses, all available cases were considered (Available Case Analysis), using data from 7,967 respondents.

To assess frequency, participants were asked how often in the past four weeks they had taken a nap or lain down around midday without doing anything active. The response categories were: ‘Every day’, ‘5 – 6 times a week’, ‘3 – 4 times a week’, ‘1 – 2 times a week’ and ‘Rarely or never’. Thus, not only afternoon naps but also periods of rest at midday were captured. As people have a limited ability to clearly distinguish between the transition from wakefulness to light sleep [11], rest periods and daytime naps were assessed together to account for this limitation. For better readability, the terms ‘daytime nap’, ‘nap’ and ‘napping’ are used interchangeably throughout the following text.

To assess the duration, all participants, except those who stated they took an ‘Afternoon nap less often or never’, were asked how long their afternoon nap usually lasts. For the analyses, the response categories ‘Up to 15 minutes’, ‘15 – 30 minutes’, ‘30 – 60 minutes’, ‘60 – 90 minutes’ and ‘More than 90 minutes’ were combined into a dichotomous variable (< 60 minutes vs. ≥ 60 minutes). A daytime nap lasting 60 minutes or more is described as an extended nap or a long nap.

The frequency of naps per week is reported as a weighted percentage of all respondents, while the proportion of respondents taking an extended nap is reported as a weighted percentage of those who report napping at least once a week (in each case with 95 % confidence intervals, 95 % CI). In all gender-stratified analyses, the data were analyzed separately according to the indicated gender identity [12]. Individuals who did not identify as either female or male were excluded from these stratified analyses due to the small sample size (n = 44). Furthermore, the data is stratified according to four age groups (18 – 29 years, 30 – 44 years, 45 – 64 years, 65 years and older) and formal educational attainment levels as defined by CASMIN (Comparative Analysis of Social Mobility in Industrial Nations [13]).

All analyses were carried out using R version 4.5.1 and RStudio version 2026.1.2.418 [14], utilizing survey procedures for complex samples. Weighting factors were used to correct for deviations within the study sample from the German population with regard to sex, age, federal state (as of 31 December 2020) and formal educational attainment [15]. Adjusted Wald tests were carried out for all reported group comparisons. Differences were considered statistically significant if the p-value was less than 0.05.

## 3. Results

In Germany, 45.3 % of adults nap at least once a week. Among women the proportion is 47.0 %, and among men it is 43.7 %, although the gender difference is not statistically significant (Figure 1). Most people who take a nap do so once or twice a week, followed by those who nap daily. Further analyses show that, in particular, people aged 65 years and older (women 26.2 %, 95 % CI: 23.0 – 29.7; men 29.2 %, 95 % CI: 25.5 – 33.2) and those with low levels of formal education (women 24.0 %, 95 % CI: 19.9 – 28.7; men 16.9 %, 95 % CI: 13.4 – 21.1) nap on a daily basis (data not shown). 

Approximately a quarter of those who take a nap at least once a week take a long nap, with no statistically significant gender difference. The lowest prevalence of extended napping is found among women and men in the oldest age group (65 years and older) (Table 1). Women in the age groups under 45 years show a higher prevalence for extended napping. However, the difference is statistically significant only for the 30 – 44 years age group compared with women aged 45 years and older, and for the 18 – 29 year age group compared with those aged 65 years and older. These differences across age groups are also present among men, although they are not statistically significant. Among men, the proportion of those taking a long nap at least once a week is statistically significantly lower in the group with a high level of formal education than in the group with a medium or low level of formal education.

## 4. Discussion

Less than half of adult women and men in Germany nap at least once a week, aligning with findings from national and international research [2, 7]. Of these, around a quarter nap at least 60 minutes. A regional German cohort study reports a lower prevalence of long nap durations at 15.6 %. However, this relates to individuals aged 45 to 75 years who take a daytime nap at least five times a week, limiting direct comparison [16]. Furthermore, the findings of the present survey show no statistically significant gender differences in terms of either nap frequency or nap duration. This is consistent with the findings of the few studies that have examined gender differences in this context [1, 3, 8]. The analysis of nap duration stratified by age group showed that women in the age groups under 45 years more frequently take an extended nap at least once a week. The increased burden of caregiving responsibilities and the resulting reduction in nighttime sleep could offer a possible explanation [17]. The significantly lower prevalence of extended daytime naps among men with high levels of formal education may be explained by the fact that men with low levels of formal education are more likely to engage in shift work, which disrupts nighttime sleep [18, 19]. Furthermore, long daytime naps among people with low levels of formal education could also result from poorer overall health, as this group is more frequently affected by health problems [20]. Among women, however, this association between formal education levels and the frequency of long daytime naps is not observed. This may be due to the fact that women across all educational levels carry an above-average burden of unpaid care work and are more frequently employed part-time or outside the workforce, potentially weakening the connection between education, employment and sleep patterns [21]. Since both age and educational attainment are associated with differences in the prevalence of long daytime naps which may be explained by variations in nighttime sleep, further analyses should investigate this relationship. Furthermore, the extent of employment should be examined more closely, as it is likely that taking a nap, and in particular an extended nap, is less frequently possible for those in full-time employment.

When interpreting the results, it should be noted that the questionnaire item used to assess the frequency of naps does not allow for a clear distinction between actual sleep and a period of inactivity spent lying down at midday. Consequently, the duration of daytime naps reported here represents only a very rough initial estimate. Additionally, the German term *'Mittagsschlaf'* used in the questionnaire item translates to midday sleep. Therefore, it cannot be determined whether respondents interpreted it as daytime sleep or specifically as midday sleep, although the term 'Mittagsschlaf' is colloquially used to describe daytime sleep regardless of the exact time. Future research should examine whether broader terms such as *‘Tagschlaf’* (daytime sleep) that do not specify a time of day are easily understood by respondents. This could ensure better comparability with internationally standardized terms such as *‘napping’*. In addition, the health impacts of inactive time spent in bed or resting as opposed to actual sleep have rarely been systematically investigated to date. This lack of research further complicates the substantive interpretation of this missing differentiation in the survey item. However, even objective measurement tools reach their limits here. For example, sedentary behavior and sleep can hardly be distinguished even with accelerometers, as these devices only capture posture and activity levels [22]. Furthermore, the results may be subject to confounding or health-related bias, as it cannot be verified whether extended napping is attributable to an underlying health condition [1]. 

Almost half of the population naps at least once a week, with a quarter of them doing so for an extended duration of 60 minutes or more. Given the positive health effects of napping, but also the potential negative effects of excessive duration, as well as the currently insufficient data situation in Germany, napping should receive greater consideration in future public health monitoring.

## Figures and Tables

**Figure 1:**
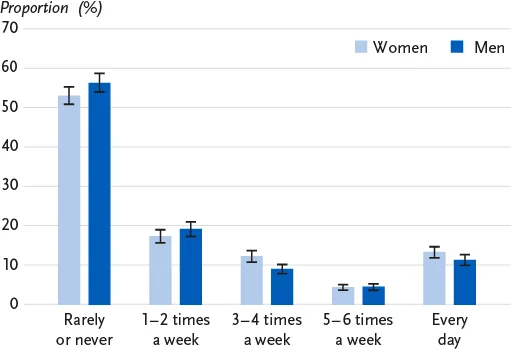
Frequency of daytime napping among the adult population in Germany by gender (n = 4,261 women, n = 3,706 men). Source: GEDA 2023

**Table 1:** Prevalence (95 % CI) of extended daytime naps (≥ 60 minutes) among those who nap at least once per week, by gender (n = 2,024 women, n = 1,772 men), age group and level of formal education. Source: GEDA 2023

	**%**	**(95 % CI)**	**%**	**(95 % CI)**
**Total (women and men)**	**25** **.** **5**	**(** **23** **.** **1** ** – ** **27** **.** **9** **)**		
	**Women**		**Men**	
**Total**	24.3	(21.3 – 27.7)	26.7	(23.3 – 30.4)
**Age groups**				
18 – 29 years	30.8	(20.9 – 42.7)	24.2	(15.7 – 35.4)
30 – 44 years	31.9	(24.1 – 41.0)	32.5	(23.4 – 43.1)
45 – 64 years	21.4	(16.7 – 27.0)	29.3	(23.1 – 36.3)
≥ 65 years	19.9	(16.2 – 24.3)	22.2	(18.0 – 27.1)
**Formal educational attainment (CASMIN)**				
Low	22.5	(17.0 – 29.3)	32.3	(25.0 – 40.6)
Medium	26.4	(22.2 – 31.1)	27.9	(22.9 – 33.4)
High	19.9	(15.3 – 25.5)	15.5	(12.3 – 19.3)

CI = confidence interval, CASMIN = Comparative Analysis of Social Mobility

in Industrial Nations
